# The 5-year outcomes of a health-empowerment program on low-income children’s behaviors and quality of life

**DOI:** 10.1186/s13034-024-00834-9

**Published:** 2024-11-10

**Authors:** Fangcao Lu, Carlos King Ho Wong, Emily Tsui Yee Tse, Amy Pui Pui Ng, Lanlan Li, Laura Bedford, Daniel Yee Tak Fong, Patrick Ip, Cindy Lo Kuen Lam

**Affiliations:** 1 Department of Family Medicine and Primary Care, Li Ka Shing Faculty of Medicine, The University of Hong Kong, 3/F, Ap Lei Chau Clinic, 161 Main Street, Ap Lei Chau, Hong Kong SAR, China; 2 Department of Applied Social Sciences, Faculty of Health and Social Sciences, The Hong Kong Polytechnic University, Hong Kong SAR, China; 3 Department of Pharmacology and Pharmacy, Li Ka Shing Faculty of Medicine, The University of Hong Kong, Hong Kong SAR, China; 4 Laboratory of Data Discovery for Health (D24H), Hong Kong Science and Technology Park, Hong Kong SAR, China; 5 Department of Family Medicine, The University of Hong Kong-Shenzhen Hospital, Shenzhen, China; 6 School of Nursing, Li Ka Shing Faculty of Medicine, The University of Hong Kong, Hong Kong SAR, China; 7 Department of Paediatrics and Adolescent Medicine, Li Ka Shing Faculty of Medicine, The University of Hong Kong, Hong Kong SAR, China; 8 Department of Paediatrics and Adolescent Medicine, Hong Kong Children’s Hospital, Hong Kong SAR, China

**Keywords:** Problematic behaviors, Psychosocial health, Child development, Health inequalities

## Abstract

**Background:**

This study aims to evaluate the 5-year impact of a Health Empowerment Program (HEP) on mitigating problematic conducts and enhancing the health-related quality of life (HRQOL) among children living in poverty.

**Methods:**

A prospective cohort study (*N* = 239, Intervention group:* n* = 124, Comparison group:* n* = 115) was established with participants recruited between July 2013 and March 2016 and followed until November 2021. During the 5-year study period, children and their parents from the intervention group were invited to join a multi-dimensional HEP. At baseline and follow-up, both intervention and comparison groups were assessed using the Chinese Strengths and Difficulties Questionnaire (SDQ) and Chinese Child Health Questionnaire Parent Form 28 (CHQ-PF28). Multiple linear regressions were conducted to identify changes in outcome variables as the effect of the HEP.

**Results:**

Upon completion of the 5-year follow-up, children in the intervention group showed a larger decline in conduct problems (B = − 0.66, *p* <.001), hyperactivity inattention (B = − 0.67, *p* =.005), and total difficulties score (B = − 1.89, *p* =.002) of SDQ, a greater increase in prosocial behavior of SDQ (B = 0.53, *p* =.040), and more substantial enhancement in CHQ-PF28’s psychosocial summary score (B = 2.75, *p* =.017) compared to the comparison group.

**Conclusions:**

HEP is effective in mitigating behavioral problems and improving psychosocial HRQOL of children of low-income families, as evident by this 5-year cohort study.

*Trial Registration*: This study received approval (UW 12–517) from the Institutional Review Board of the University of Hong Kong/Hospital Authority Hong Kong West Cluster.

**Supplementary Information:**

The online version contains supplementary material available at 10.1186/s13034-024-00834-9.

## Background

Living in poverty poses multiple risks for children’s overall health and development [1, 2]. In addition to difficulties in meeting basic amenities, low-income families are often characterized by increased illnesses and family stresses, limited resources, and a lack of psychosocial stimulation, and these families often live in low-income neighborhoods that have poor education, more crime, and high density, which can hinder the growth and development of children [2–4]. Research shows that children living in poverty experience lower health-related quality of life (HRQOL) and a higher prevalence of behavioral problems than children in the general population [5]. Early experiences with poverty can have additional detrimental effects on children’s future physical and psychological health and achievement as adults.[6–8]. Given that poverty tends to perpetuate itself [9], efforts to develop effective interventions aimed at improving health and development among low-income children have been advocated to disrupt the cycle of intergenerational poverty and poor health outcomes [10].

Primary healthcare is seen as the key to achieving universal health coverage and addressing health inequalities [11–13]. The goal of primary healthcare is to empower individuals to gain greater control over their lives and make decisions about their health, through a process known as health empowerment, which raises their awareness of their capacity to do so [14]. Health empowerment involves supporting individuals, families, and communities to enable health knowledge, motivation, self-assurance, utilization of health resources, and self-care to maintain health [15]. A growing body of evidence has shown that an effective health empowerment program (HEP) can help adolescents develop greater confidence and self-control over their behavior and health-related actions, and reduce mental health problems as well as problematic, impulsive, and pathological behaviors [16–18]. For instance, an 8-week empowerment program in Australia, which included weekly educational and practical sessions along with home tasks, delivered to both fathers and daughters, was found to improve physical activity, and enhance fundamental movement skill proficiency, perceived family relationships, and socio-emotional well-being in daughters [19, 20]. Additionally, a 6-week collaborative intervention in Korea, which involved discussing mutually agreed-upon goals, visualizing and explaining shared planning, sharing family routines and activity matrices, and discussing changes in child performance, was found to improve parents’ empowerment and child performance [21]. Nevertheless, there is limited evidence regarding the effectiveness of health empowerment interventions in the Chinese population, which constitutes a significant portion of the world’s population. Additionally, evidence of the ability of these interventions to produce positive changes in young children from impoverished households is also scarce.

The current study was undertaken in Hong Kong, which ranks among the top regions with the highest Gini coefficients at 0.54, indicating a wide economic inequality [22]. According to the local criteria of poverty (< 50% of population median income), more than 20% of the population in Hong Kong (1.65 million people) live in poverty [23]. Hong Kong has a high cost of living and private medical costs [24] coupled with limited public assistance can cause people from such low-income families to struggle with their basic living and health needs. Tung Chung, a new town in Hong Kong, covers approximately 200 hectares on a remote island [25]. According to the 2011 census (two years preceding our study), Tung Chung had 78,000 residents [26] living in 21,695 families, with a poverty rate of 40% [27]. Household monthly income varies by district in Hong Kong. In Tung Chung North, which consists mainly of private housing, the income is 45,330 HKD. In contrast, the Yat Tung area, where public housing is predominant, has lower incomes—12,300 HKD for Yat Tung North and 18,650 HKD for Yat Tung South. These figures are below the 2011 Hong Kong average income of 20,500 HKD [28]. Our study recruited families from public housing estates in Yat Tung, which include over 13,000 residential units and make up more than half of Tung Chung’s population [29]. We focused on families with monthly household earnings not exceeding 75% of the median monthly household income of Hong Kong families, which is an eligibility criterion for public housing in Hong Kong. In 2013, when the study began, Tung Chung had only one primary care clinic and a newly opened district hospital [30], making healthcare scarce for low-income families unable to afford private treatment. Due to the absence of competition and management by private real estate investment trusts, Tung Chung has some of the highest prices in Hong Kong [31], exacerbating financial difficulties for these families living in Tung Chung. Research shows that the coexistence of poverty and inadequate public healthcare services concurrently can harm the health and development of children [32]. One local study has found that both those living in poverty and the those earning between 50 and 75% have poorer health and HRQOL compared to the general population [33].

Thus, in 2012, the Trekkers Family Enhancement Scheme (TFES) was launched to aid financially disadvantaged households from Tung Chung. The scheme aims to provide assistance in various areas, including healthcare, education, employment, and family/environmental well-being, to enhance the families’ quality of life and develop their full potential by delivering various activities organized by non-government organizations (NGO) (for details, see Supplementary Table 1). We designed and implemented a HEP to promote the health of families in TFES. Empowerment in the health context involves professionals creating opportunities and providing support so individuals and families can actively participate in health problem formulation, decision-making, and actions [34]. Thus, our HEP includes four diverse yet interrelated components, including health literacy, self-care enablement, health ambassador, and health assessment, offering support in exercise, nutrition, health assessments, cues to actions etc. Additionally, empowerment promoting children’s health recognizes families as partners in health care and respects children’s agency as active participants in family, community, and society [35]. A systematic review [35] found that empowering young children and families through participation increases the effectiveness of health interventions. Therefore, we involve not only children but their entire families with the expectation that knowledge, attitudes, and practices of healthy living can be shared among family members and peers within the community. We believe that participants can still benefit from the four components even if they do not actively attend all HEP activities. We also believe that this approach enhances generalizability, as it would be unrealistic to expect people to participate in all the intervention activities, especially those in which they have no interest. It makes more sense for them to join the activities that resonate with them. The objective of this study was to assess the 5-year impact of the HEP on the development and health of children and examine the hypothesis on whether the HEP was associated with a reduction in behavioral problems and improvement in HRQOL over a 5-year follow-up.

## Method

### Study design

This cohort study involved the comparison of two groups of families with young children in Grades 1–3 at the time of study initiation. All families in TFES were eligible and invited to participate in the HEP (referred to as "intervention families") and a comparison group was selected from families in Tung Chung and Kwai Chung who did not join the TFES. Kwai Chung is an emerging suburban community in Hong Kong with limited access to healthcare services and therefore has a similar sociodemographic background to Tung Chung [36]. Participants were recruited between July 2013 and March 2016. Families were recruited when they met the following criteria: (1) One or more family members were employed, either on a full-time or part-time basis, (2) had a child or children enrolled in a primary school program for grades 1 to 3, (3) had a monthly household earnings that did not exceed 75% of the median monthly household income in Hong Kong during that period. Each intervention and comparison family could have more than one child included in the study. Children in both groups had cognitive skills assessments by qualified clinical psychologists at enrollment. In addition, a comprehensive health evaluation and a phone survey were administered for their parents as a baseline and again after approximately 5 years as a follow-up. During the 5-year follow-up, intervention families were offered regular HEP activities and could participate if they wished. Meanwhile, families in the comparison group were not asked to participate the intervention activities.

### Components of HEP

The HEP included intercalated routine health evaluations, self-management education, health knowledge talks, and health ambassador training. Among these, the first two types of activities were available to children participants together with their parents, while the latter two types of activities targeted the parents.

#### HEP activities for both children and parents

The annual health assessment included a telephone survey about health and health service use and an in-person clinical health assessment. In the telephone survey, parents answered the survey questions about their children’s health, healthcare service utilization, behavior, and HRQOL. The clinical health assessment of children was carried out by registered nurses, trained technicians, and research assistants. Children identified as having substantial health risks or abnormalities were offered counseling by a nurse or doctor from the project team or were directed to suitable services for additional management. The yearly count of individuals facing major health issues or risks, along with the referrals they obtained, can be found in Supplementary Table 2. Self-care enablement activities (see Supplementary Table 3) included workshops and training courses on nutrition, cooking and exercise, family gym, and hiking groups in which the children participated together with their parents. Participants in these activities reported significantly improved self-care enablement after engaging in these activities. Additionally, over 90% of participants expressed satisfaction and indicated they would recommend the activities to their family and friends. The self-care components also created a mobile app, *FamilyMove*, delivered to these parents and their children to provide coaching after the face-to-face training.

#### HEP activities for parents

In addition to annual health assessments and activities available to both children and parents, recurring health lectures and talks on typical issues detected in the health evaluations were delivered to parents to enable better self-care (see Supplementary Table 4). The participants’ understanding of the health topic was assessed before and after each seminar, revealing a significant improvement. Additionally, several parents received training to serve as leaders for nutrition and physical activity classes and to manage group exercises following the classes. These adults acted as health ambassadors for their acquaintances and families.

### Outcome measures

The Chinese Strengths and Difficulties Questionnaire (SDQ) [37] was used to assess children’s behavioral issues. The SDQ is one of the most widely used questionnaire for child emotional and behavioral screening globally. The measure has been translated into over 80 languages and serves various purposes, including clinical assessment, outcome evaluation, research, and screening [38]. The SDQ was used to assess 5 domains of children’s behaviors, including emotional issues, behavioral misconduct, attention difficulties, socialization difficulties and prosocial conduct. Each of these domains has a 5-item subscale. The measure is valid and reliable in the context of Hong Kong [37]. A 3-point Likert scale, in which 0 = “did not apply,” 1 = “apply to a certain extent,” and 2 = “applied very much”, is used to record the participants’ answers. The overall score for each issue/behavior is computed by adding the scores of all items in the corresponding subscale. Thus, the score on each problem/behavior ranges from 0 to 10. The score of total difficulties is computed by adding up the scores on the four problem subscale scores [39] and ranges from 0 to 40. A greater value for the specific difficulty subscale and the total score of difficulties indicate more severe problems, while a higher score for prosocial behavior subscale is indicative of better prosocial behaviors.

The Chinese Child Health Questionnaire Parent Form 28 (CHQ-PF28) [40] was administered to assess children’s HRQOL. The CHQ-PF28 is a widely recognized pediatric health survey designed to evaluate HRQOL in children and adolescents aged 5–18. This measure has been developed and validated, demonstrating its effectiveness across various regions and populations. It has been utilized in over 600 peer-reviewed journal articles [41]. The measure has shown good validity and reliability in a Chinese setting [42]. It consists of 28 items, which cover 12 domains*,* including general health, physical functioning, emotional/behavioral limitations, physical limitations, bodily pain, general behavior, mental health, self-esteem, parent impact on emotion, parent impact on time, family activities, and family cohesion. The scores of these domains range from 0 to 100, which can be computed using a weighted summation algorithm detailed in the CHQ manual [43]. The aggregated scores comprise a physical summary score (PHS) and psychosocial summary score (PSS), which can be translated into norm-based scores where the mean score of the general population is 50, with a standard deviation of 10 [44]. A greater value indicates better HRQOL.

### Baseline covariates

#### Cognitive skills

The Wechsler Intelligence Scale for Children–Fourth Edition (WISC–IV) [45] was used to assess cognitive skills of children in both groups and the assessment was conducted by clinical psychologists. This instrument has shown good validity among Hong Kong children [46, 47]. The measure can generate an intelligence quotient (IQ) score, and a higher value represents a higher intellectual capacity.

#### Socioeconomic and health status

The following variables were also included as baseline covariates: children’s age, gender, body weight status, known doctor diagnosis of learning disability and chronic diseases, parents’ marital condition, monthly household revenue, and governmental comprehensive social security assistance (CSSA) scheme reception.

The SDQ, CHQ-PF28, and the other baseline covariates questionnaires were carried out by skilled interviewers, either in face-to-face or telephonic settings, and responses were provided by one or both parents of the children through self-reporting. The face-to-face interviews were administered by trained Research Assistants (RAs), and registered nurses. All interviewers received training from the project investigators and registered nurses on health assessment and survey administration, and their performance was monitored by the nurses during the initial data collection sessions. The telephone interviews were conducted by the Social Science Research Centre (SSRC) of the University of Hong Kong, a research center with over 10 years of experience in telephone surveys. SSRC staff received training in social science research methods, including phone survey techniques. All interviews adhered strictly to the interview protocol.

### Statistical analysis

We used intention to treat and complete-case analysis in that all participants with baseline and follow-up assessments being included in the analysis. Stata version 16.0 (StataCorp LP, College Station, Texas) was employed for all statistical analyses. Statistical significance was determined using two-tailed tests, with *p* < 0.05 as the threshold for significance.

Descriptive statistics were employed to display the baseline characteristics of the participants. The *p*-value was used to evaluate the balance of baseline covariates between the two groups, where *p* > 0.05 indicated that the two groups were significantly different from each other. The change in each measure between baseline and follow-up was computed by subtracting the baseline score from the follow-up score. The statistical significance of the within-group change was assessed via paired samples *t*-test, and the effect size was calculated by Cohen’s d.

Multiple linear regressions were performed to investigate the independent impact of HEP on the changes in SDQ and CHQ-PF28 scores after 5-year follow-up, adjusting for covariates and baseline values. For each model, F-ratio and significance of the F-ratio were used to indicate the extent to which the predictors can explain the variance in the outcome variables in a statistically significant manner. The adjusted R^2^ was used to indicate the extent to which the variance in the predictor variances (as a whole) explains the variance in the outcome variable. Also, unstandardized B, *p*-value, and 95% confidence level were determined to show the influence of HEP on each outcome measure. Bonferroni correction was performed. The threshold for significance was adjusted by dividing the alpha level by the number of analyses, resulting in a threshold of 0.008 for the SDQ analyses and 0.025 for the CHQ-PF28 analyses. To provide a more detailed investigation, we repeated the above analyses to examine the effect of the HEP on changes in each of the 12 domains of the CHQ-PF28.

Subgroup analyses were further conducted based on baseline covariates. These analyses aimed to identify which groups benefited most from the intervention and to understand the impact of the intervention across different sociodemographic groups. Additionally, to evaluate the robustness of the study findings, a sensitivity analysis was performed by further adjusting for mothers’ sociodemographic and health status in addition to baseline covariates and values in multiple linear regressions. In addition, generalized estimating equations with exchangeable correlation structures were applied to account for the correlations of outcomes between children belonging to the same family in addition to baseline covariates and values.

## Results

**Fig. 1:**
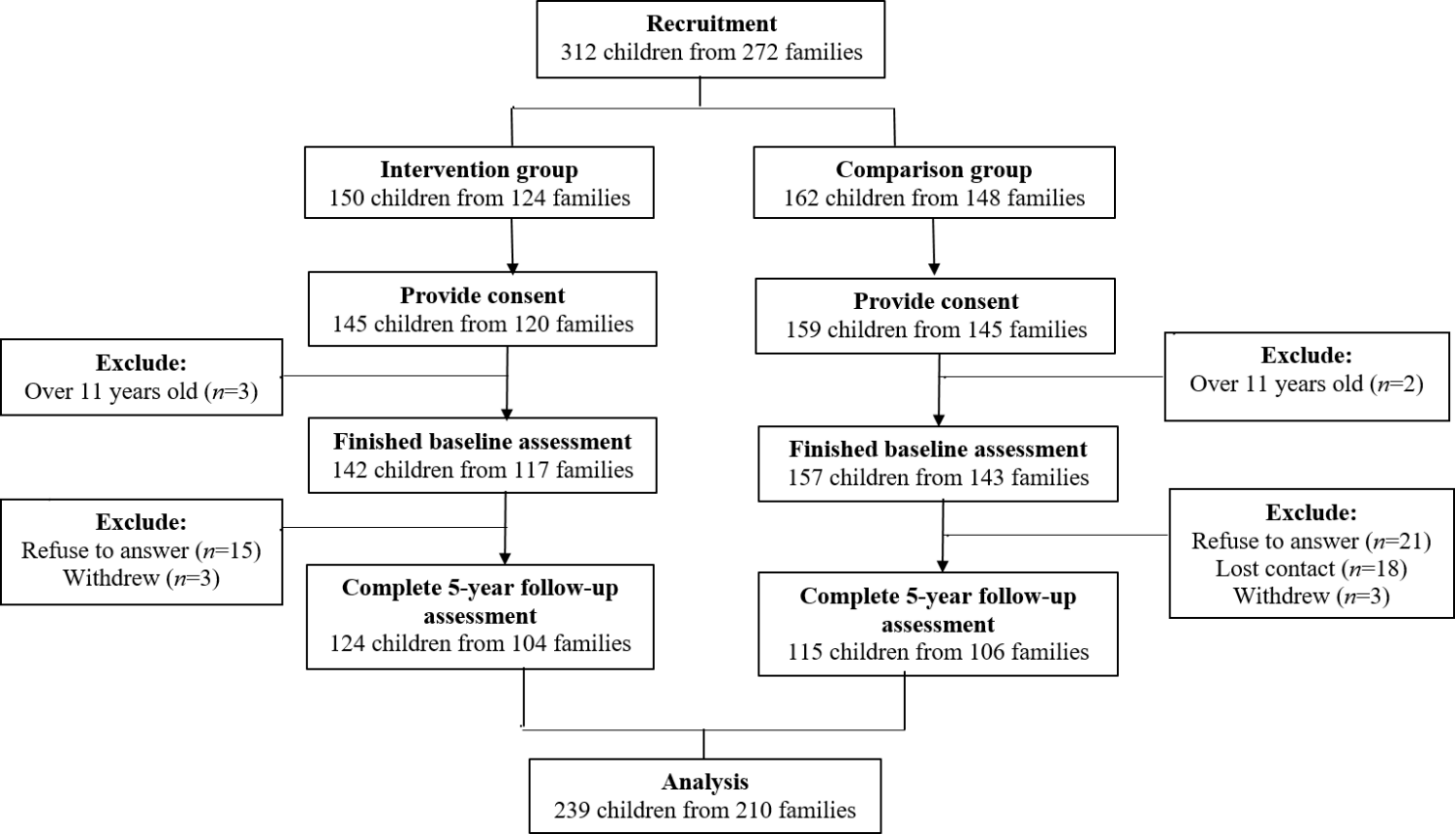
Subject flowchart

In all, 312 children-parent pairs were recruited from July 2013 to March 2016, and 301 children-parent pairs consented and finished the baseline evaluation. Sixty children-parent pairs (42 pairs within comparison group and 18 within intervention group) did not finish the follow-up evaluation. Most of these children were aged between 8 and 11 years old (70%, *n* = 42), were male (51.67%, *n* = 31), and did not have a chronic disease (87.72%, *n* = 50). The majority of their parents were married (66.67%, *n* = 32) and had a family monthly income lower than 20,000 HKD (93.88%, *n* = 46). Finally, 239 eligible children-parent pairs from 210 families with an average follow-up period of 5 years were included in the analysis. Among 210 families, 181 families had only one child, and of the rest, 29 families had two children. The follow-up period for the intervention group ranges from 42 to 96 months, and that in the comparison group ranges from 42 to 92 months. Among the participants in the comparison group, 29.6% of them (*n* = 34) were recruited from Tung Chung whereas 70.4% of them (*n* = 81) were recruited from Kwai Chung. The flow of the subject’s recruitment and follow-up were presented in Fig. 1.

### Participant characteristics

The baseline features of the 239 eligible children and their families are illustrated in Table 1. All baseline characteristics were balanced between the intervention and comparison groups, as indicated by all *p* > 0.05. In the intervention and comparison groups, the average age of the children was 8.59 and 8.23, respectively. Overall, 16.6% of children had chronic ailments, and the most prevalent diagnoses were asthma (*n* = 13), anemia (*n* = 4), and mental illness (*n* = 2). Learning disabilities were reported in 13.3% of the children. The mean IQ score was 100 (SD = 14) for the intervention and 101 (S.D = 14) for comparison groups. As for family socioeconomic status, most children (85.8%) had married parents. The mean of their family monthly income was 14,458.3 HKD (SD = 7204.9HKD), which was lower than the median monthly household revenue (22,000 HKD) of the overall Hong Kong population in 2013 [48]. Additionally, about 23.5% of families received government CSSA. The baseline characteristics of the mother were listed in Supplementary Table 5. Overall, most mothers had normal weight (64.86%), with baseline chronic disease (60.81%), and had secondary or above education (92.03%).

**Table 1:** Baseline characteristics

Children socioeconomic status				
	Total	Intervention	Comparison	*p-value*
Age, years	*N* = 239	*N* = 124	*N* = 115	0.530
	8.41 ± 1.14	8.59 ± 1.23	8.23 ± 1.00	
6–7	56 (23.43%)	27 (21.77%)	29 (25.22%)	
8–11	183 (76.57%)	97 (78.23%)	86 (74.78%)	
Gender	*N* = 239	*N* = 124	*N* = 115	0.179
Male	123 (51.46%)	69 (55.65%)	54 (46.96%)	
Female	116 (48.54%)	55 (44.35%)	61 (53.04%)	
Body weight status	*N* = 235	*N* = 122	*N* = 113	0.151
Underweight	3 (1.28%)	3 (2.46%)	0 (0.00%)	
Normal	180 (76.60%)	89 (72.95%)	91 (80.53%)	
Overweight	33 (14.04%)	17 (13.93%)	16 (14.16%)	
Obese	19 (8.09%)	13 (10.66%)	6 (5.31%)	
IQ level	*N* = 239	*N* = 124	*N* = 115	0.586
	100.45 ± 14.00	99.98 ± 14.10	100.97 ± 13.93	
Chronic disease	*N* = 235	*N* = 122	*N* = 113	0.662
Yes	39 (16.60%)	19 (15.57%)	20 (17.70%)	
No	196 (83.40%)	103 (84.43%)	93 (82.30%)	
Learning disability	*N* = 234	*N* = 121	*N* = 113	0.991
Yes	31 (13.25%)	16 (13.22%)	15 (13.27%)	
No	203 (86.75%)	105 (86.78%)	98 (86.73%)	
Parental marital status	*N* = 239	*N* = 124	*N* = 115	0.111
Married	205 (85.77%)	112 (90.32%)	8 (6.96%)	
Divorce	22 (9.21%)	8 (6.45%)	93 (80.87%)	
Single	12 (5.02%)	4 (3.23%)	14 (12.17%)	
Family monthly income ($HKD)	*N* = 238	*N* = 124	*N* = 114	0.606
	14,458.3 ± 7204.9	14,226.8 ± 6253.1	14,710.1 ± 8135.7	
Government CSSA	*N* = 234	*N* = 119	*N* = 115	0.993
Yes	55 (23.50%)	28 (23.53%)	27 (23.48%)	
No	179 (76.50%)	91 (76.47%)	88 (76.52%)	

*IQ* Intelligence quotient*. CSSA* comprehensive social security assistance.

### Changes in SDQ and CHQ-PF28 Scores

The SDQ and CHQ-PF28 scores of both groups in baseline and follow-up assessments are displayed in Table 2. Additionally, supplementary Tables 6 and 7 summarize the changes in scores across each of the 12 domains within the CHQ for the total study population and the two study groups, respectively.

**Table 2:**
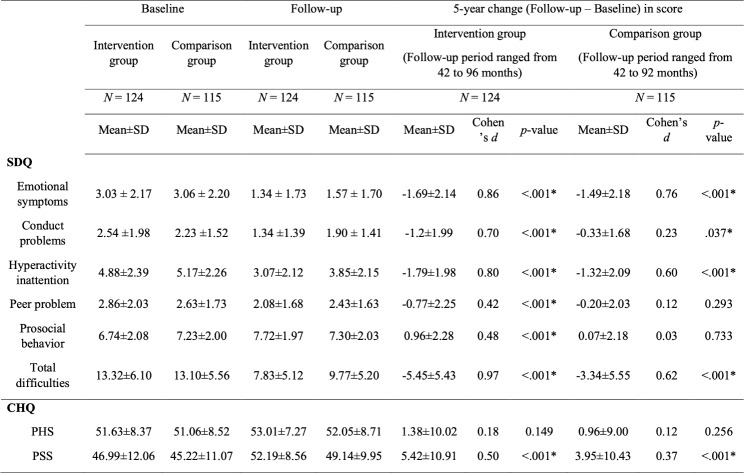
Comparison of changes in SDQ and CHQ between intervention groups and comparison group

*SDQ* Strengths and Difficulties Questionnaire, *CHQ* Child Health Questionnaire Parent Form 28, *PHS* Physical Summary score, *PSS* Psychosocial Summary score

*p value by paired sample t test, Cohen’s effect size d = difference between baseline and follow-up scores/pooled standard deviations of the two scores

The results of* t*-test (Table 2) showed that among children in the intervention group, there were significant decreases (all *p* < 0.001, Cohen’s *d* >  = 0.42) in all behavior problems and total difficulties score of the SDQ and an increase (*p* < 0.001, Cohen’s *d* = 0.48) in prosocial behavior score of SDQ over the 5-year follow-up. Children from the intervention group also experienced a significant increase (*p* < 0.001, Cohen’s *d* = 0.50) in the CHQ PSS score, and a small increase in CHQ PHS score that was not statistically significant (*p* = 0.149). Additionally, the two supplementary tables showed that over the 5-year follow-up period, the scores in domains of role/social-emotional/behavioral, behavior, mental health, physical functioning, parent impact–emotion, and family activities significantly increased in the intervention group (all *p* < 0.05).

Children from the comparison group experienced a decrease in emotional (*p* < 0.001, Cohen’s *d* = 0.76), conduct (*p* = 0.037, Cohen’s *d* = 0.23), hyperactivity problems (*p* < 0.001, Cohen’s *d* = 0.60), and total difficulties (*p* < 0.001, Cohen’s *d* = 0.62) scores of SDQ. The decrease in peer problems (*p* = 0.293) and increase in prosocial behavior (*p* = 0.733) of SDQ were not significant. The children from the comparison group also experienced an increase in CHQ PSS score (*p* < 0.001, Cohen’s *d* = 0.37), while their increase in CHQ PHS scores was not significant (*p* = 0.256). The effect sizes of the change in the SDQ and CHQ scores were generally smaller in the comparison group compared to the intervention group. Additionally, the two supplementary tables showed that over the 5-year follow-up period, the scores in domains of role/social-emotional/behavioral, bodily pain/discomfort, behavior, physical functioning, parent impact—emotion, self-esteem, and family activities significantly increased in the comparison group (all *p* < 0.05).

### The outcomes of HEP on 5-year changes in SDQ and CHQ

The results of the multiple linear regression of the health intervention on the changes in SDQ and CHQ scores are displayed in Table 3. Each row in Table 3 represents a separate multiple regression analysis, with participation in the HEP as the independent variable and changes in each domain as the outcome variable. The analysis also controls for the aforementioned covariates and baseline values. The results show that children from the intervention group experienced greater decreases in behavioral problems measured by the SDQ scores, including those with behavioral misconduct (B = − 0.66, *p* < 0.001), attention difficulties (B = − 0.67, *p* = 0.005), as well as total difficulty (B = − 1.89, *p* = 0.002). Moreover, the HEP was linked to a larger rise in CHQ PSS score (B = 2.75, *p* = 0.017). Multiple linear regression analyses (Supplementary Table 8) reveal that participation in the HEP was associated with a larger increase in physical functioning (B = 5.42, *p* = 0.041), behavior (B = 7.44, *p* = 0.002), and parent impact– emotion (B = 7.22, *p* = 0.008).

**Table 3:** Association between health empowerment program and changes in outcomes after 5-year follow-up

	B (95% CI)	*p*-value for *β*	*Adjusted R*^*2*^	*F ratio*		*p value for F ratio*
SDQ						
Emotional symptoms	− 0.11 (− 0.54, 0.32)	0.607	0.47	15.46	< 0.001*	
Conduct problems	− 0.66 (− 1.01, − 0.31)	< 0.001*	0.52	18.33	< 0.001*	
Hyperactivity inattention	− 0.67 (− 1.14, − 0.20)	0.005*	0.27	7.04	< 0.001*	
Peer problem	− 0.34 (− 0.78, 0.10)	0.126	0.45	14.45	< 0.001*	
Prosocial behavior	0.53 (0.02, 1.03)	0.040	0.34	9.17	< 0.001*	
Total difficulties	− 1.89 (− 3.10, − 0.68)	0.002*	0.35	9.76	< 0.001*	
CHQ						
PHS	0.74 (− 1.43, 2.91)	0.503	0.38	8.78	< 0.001*	
PSS	2.75 (0.49, 5.02)	0.017*	0.42	12.05	< 0.001*	

*SDQ* Strengths and Difficulties Questionnaire, *CHQ* Child Health Questionnaire Parent Form 28, *PHS* Physical Summary score, *PSS* Psychosocial Summary score.

*Bonferroni correction was performed. For SDQ analyses, the threshold for significance is reduced to alpha/number of analyses = 0.05/6 = 0.008; *p* value < 0.008 is statistically significant; For CHQ analyses, the threshold for significance is reduced to alpha/number of analyses = 0.05/2 = 0.025; *p* value < 0.025 is statistically significant. Aforementioned covariates and baseline scores in the respective domain is controlled for in the analysis.

Subgroup analyses show similar results among most subgroups across different domains within SDQ and CHQ (Supplementary Table 9). The association between the HEP and peer problems significantly interacted with age group (*p* = 0.005) and chronic diseases (*p* = 0.011). The results indicated that compared to children aged 8–11 years, children aged 6–7 years were more likely to experience a reduction in peer problems after participating in our program. Additionally, children without chronic diseases were more likely to reduce peer problems compared to those with chronic diseases after participation. Furthermore, the association between HEP and prosocial behavior significantly interacted with age group (*p* = 0.026). Children aged 6–7 years were more likely to exhibit prosocial behavior compared to those aged 8–11 years. Moreover, we found that the association between HEP and the PHS score interacted with gender (*p* = 0.024). The intervention was associated with a significant increase in PHS scores among males but not females.

The two sensitivity analyses (Supplementary Table 10 and 11) consistently showed significant associations between HEP and a greater decrease in the SDQ behavioral problems scores, a greater increase in the SDQ prosocial behavior score, and a greater increase in CHQ PSS score.

## Discussion

This study is one of the earliest to examine the impact of a complex health empowerment program on the development and HRQOL of children living in poverty. The results show that HEP was associated with a significant reduction of problematic behaviors, and improvements in prosocial behaviors and HRQOL in children of low-income families.

Earlier research has mainly investigated the impact of diverse HEPs in enhancing adults’ health-related outcomes [49] and the evidence of the impact of HEP on minors is limited to teenagers [17, 18]. The findings in this study affirmed the benefit of the longitudinal HEP for young children living in poverty. The most significant benefit was in reducing children’s problematic behaviors, increasing their prosocial behavior, and increasing self-report psychosocial HRQOL. Many of our HEP activities promoted physical activities, which not only drive biopsychosocial mechanisms to change the structural composition of the brain but also offer opportunities for social interaction and increase confidence in children [50, 51]. The health assessment serves as a form of social support for children and their parents, which enables them to take greater charge of their health and address any potential problems [52]. The HEP also improved the parents’ health literacy, parenting practice, and family harmony, which are critical for children’s development [53]. The perceived self-control over health or life-related actions and the enhanced confidence and positive feedback ensuring good performance all contributed to the inhibition of negative emotions [54] and therefore increased HRQOL.

No significant relationship was found between HEP and a change in physical HRQOL in either group in the 5-year follow-up period. The COVID-19 outbreak, which occurred toward the conclusion of the intervention, may have influenced the study’s intervention effect. The Hong Kong government implemented regulations during the peak of the outbreak in 2020, which involved the closure of indoor fitness centers and restrictions on outdoor exercise lasting over five months [55]. These measures may have resulted in restricted physical movements, which could have negatively impacted physical wellness, ultimately affecting physical ability and overall health, the significant determinants of the CHQ PHS. Furthermore, previous studies also found limited effectiveness of HEP in improving children’s physical functioning. The authors in these studies explained that in addition to the problem of intervention design (i.e., disorderly discussion organization) [56], another possible explanation was that the comparison group may have been affected by the assessment procedure that might have motivated them to start exercising without intervention[57].

The present study provides theoretical and policy implications. Initially, fostering health empowerment is a feasible mean by which to escape the trap of poverty and ill health through enhanced health literacy and self-care. Second, we not only involved children and their parents in the routine health evaluation and self-care enablement education, but also facilitated appropriate primary healthcare to address problems early, and provided health ambassador training to the parents. It suggests the importance of family participation and the reciprocal impact within families when designing complex empowerment interventions. Third, our HEP included diverse yet interconnected elements to improve not just health literacy and behavior, but also equitable access to medical and social services for addressing health and social challenges. These distinct components provide valuable lessons for developing complex empowerment interventions in practical contexts. Fourthly, we assessed and controlled for children’s IQ levels in the analysis to ensure effectiveness in improving health and development for children were independent of intelligence. Finally, for policymakers, empowering and involving children in the process and recognizing families as partners in empowerment are feasible approaches to reducing disparities among children from low-income households.

There are potential limitations. First, there could have been a selection bias as individuals in the HEP enrolled voluntarily and may have had preexisting motivation to seek health-related knowledge and support compared to others. Allocation of the participant into an intervention or comparison group was not randomized and this could be a major bias to the results from unknown confounders. Furthermore, the intervention was confined to individuals presently living in a particular area of Hong Kong, so it may not be a representative sample and thus the results may not be generalizable to children living in other districts. Nonetheless, it is essential to point out that the baseline covariates between the groups were similar, and the covariates were adjusted in the regression analysis to control for residual confounding bias. Also, we conducted a sensitivity analysis that adjusted for the mothers’ sociodemographic and health status, and the results were consistent with our main analysis. We encourage future studies to randomize participants into different conditions to enhance the robustness of their findings.

Second, the data on outcome variables were self-reported by parents, which could have the potential to bias from a tendency to give socially acceptable answers. However, this was equally relevant for both the intervention and comparison groups and for both the baseline and follow-up data. Third, TFES is a complex intervention that involves families receiving employment and education support in addition to the HEP. The other aspects of TFES could impact scores on CHQ and SDQ.

Finally, the findings from the present study are solely determined by parent interviews, which may cause bias. Our approach considers the age range of the children in this study, who are between 6 and 11 years old. While children aged 10 or 11 may be capable of understanding survey questions and providing answers based on their personal experiences, younger children aged 6 or 7 may not have fully developed reading and comprehension skills. Interviewing or asking these younger children to complete questionnaires could result in biased data due to their incomplete language development. Therefore, we chose to use parent interviews rather than children’s self-reports. We suggest that future studies adopt a multi-informant approach. For example, teachers could be invited to provide assessments, which could then be combined with parents’ data to offer a more comprehensive representation of children’s behavior and quality of life. Additionally, future research could develop questionnaires that are easily understood by children, allowing for their self-reports to be combined with parents’ data to provide a more complete picture.

## Conclusions

This study proves the benefits of a longitudinal HEP in reducing behavioral problems and enchaining psychosocial HRQOL for children living in poverty, demonstrating its potential to mitigate health disparities among children from low-income households. We involved both children and their parents in recognition of the reciprocal impact of family members on each other’s health. The diverse yet interconnected components provide valuable lessons for developing complex empowerment interventions in practical contexts. This creates a new area of research to explore how HEP care models can be more extensively adopted.

## Supplementary Information

Additional file1 (DOCX 79 kb)

## Data Availability

The data is available upon contacting the corresponding author.

## References

[1] Kalil A, Duncan GJ, Ziol-Guest KM. Early childhood poverty: Short and long-run consequences over the lifespan. Handbook of the Life Course. 2016;2:341–54.

[2] Spencer N: *Poverty and child health.* CRC Press; 2018.

[3] Larson K, Halfon N. Family income gradients in the health and health care access of US children. Matern Child Health J. 2010;14:332–42. 19499315 10.1007/s10995-009-0477-y PMC2862175

[4] Engle PL, Black MM. The effect of poverty on child development and educational outcomes. Ann N Y Acad Sci. 2008;1136:243–56. 18579886 10.1196/annals.1425.023

[5] Wong RSM, Guo VY, Ip P, Wong CKH, Yu EYT, Fung CSC, Lam CLK. Mothers’ health-related quality of life: Its relationship with children’s health-related quality of life and behavior in low-income families. Family Medicine and Community Health. 2016;4:4–12.

[6] Black MM, Walker SP, Fernald LC, Andersen CT, DiGirolamo AM, Lu C, McCoy DC, Fink G, Shawar YR, Shiffman J. Early childhood development coming of age: science through the life course. The Lancet. 2017;389:77–90. 10.1016/S0140-6736(16)31389-7 PMC5884058 27717614

[7] Miller GE, Chen E, Parker KJ. Psychological stress in childhood and susceptibility to the chronic diseases of aging: moving toward a model of behavioral and biological mechanisms. Psychol Bull. 2011;137:959. 21787044 10.1037/a0024768 PMC3202072

[8] National Research Council: From neurons to neighborhoods: The science of early childhood development. 2000. 25077268

[9] Kraay A, McKenzie D. Do poverty traps exist? Assessing the evidence. Journal of Economic Perspectives. 2014;28:127–48.

[10] Lake A. Early childhood development—global action is overdue. The Lancet. 2011;378:1277–8. 10.1016/S0140-6736(11)61450-5 21944376

[11] World Health Organization: Primary health care: report of the International Conference on primary health care, Alma-Ata, USSR, 6–12 September 1978. World Health Organization; 1978.

[12] Van Lerberghe W: *The world health report 2008: primary health care: now more than ever.* World Health Organization; 2008.

[13] Primary health care. https://www.who.int/health-topics/primary-health-care#tab=tab_1

[14] Talbot L, Verrinder G: *Promoting health: the primary health care approach.* Elsevier Health Sciences; 2017.

[15] Anderson JM. Empowering patients: issues and strategies. Soc Sci Med. 1996;43:697–705. 8870134 10.1016/0277-9536(96)00153-0

[16] Damen H, Scholte RH, Vermulst AA, Van Steensel P, Veerman JW. Parental empowerment as a buffer between parental stress and child behavioral problems after family treatment. Child Youth Serv Rev. 2021;124: 105982.

[17] Ghahremani DG, Oh EY, Dean AC, Mouzakis K, Wilson KD, London ED. Effects of the Youth Empowerment Seminar on impulsive behavior in adolescents. J Adolesc Health. 2013;53:139–41. 23601502 10.1016/j.jadohealth.2013.02.010

[18] Sarkar K, Dasgupta A, Sinha M, Shahbabu B. Effects of health empowerment intervention on resilience of adolescents in a tribal area: A study using the Solomon four-groups design. Soc Sci Med. 2017;190:265–74. 28625414 10.1016/j.socscimed.2017.05.044

[19] Morgan PJ, Young MD, Barnes AT, Eather N, Pollock ER, Lubans DR. Engaging fathers to increase physical activity in girls: the “dads and daughters exercising and empowered”(DADEE) randomized controlled trial. Ann Behav Med. 2019;53:39–52. 29648571 10.1093/abm/kay015

[20] Young MD, Lubans DR, Barnes AT, Eather N, Pollock ER, Morgan PJ. Impact of a father–daughter physical activity program on girls’ social–emotional well-being: A randomized controlled trial. J Consult Clin Psychol. 2019;87:294. 30640483 10.1037/ccp0000374

[21] An M, Palisano RJ, Yi C-H, Chiarello LA, Dunst CJ, Gracely EJ. Effects of a collaborative intervention process on parent empowerment and child performance: A randomized controlled trial. Phys Occup Ther Pediatr. 2019;39:1–15. 28929830 10.1080/01942638.2017.1365324

[22] The Gini Coefficient of Hong Kong’s Inequality https://www.lwb.gov.hk/tc/blog/post_08082021.html

[23] Government of the Hong Kong Special Administrative Region: Hong Kong Poverty Situation Report 2020. 2021. https://www.commissiononpoverty.gov.hk/eng/pdf/Hong_Kong_Poverty_Situation_Report_2020.pdf

[24] Worldwide Cost of Living 2022. https://www.eiu.com/n/campaigns/worldwide-cost-of-living-2022/#:~:text=Global%20prices%20have%20risen%20by,expensive%20cities%20in%20the%20world

[25] Tung Chung: Planning for Liveable New Towns https://www.pland.gov.hk/pland_en/outreach/educational/NTpamphlets/pdf/nt_is_tc.pdf

[26] Census and Statistics Department GotHKSAR: 2011 Population Census - Main Tables (New Town). 2011. https://www.censtatd.gov.hk/en/EIndexbySubject.html?scode=170&pcode=D5211108

[27] The Hong Kong Council of Social Service: Low Income Household Population in outlying island of Hong Kong Report.; 2006. https://webcontent.hkcss.org.hk/pra/report_card/Islands.pdf

[28] Tung Chung New Town Expansion Study - Supplementary information provided at the request of Members https://www.legco.gov.hk/yr14-15/chinese/panels/dev/papers/dev20141203cb1-1132-1-c.pdf

[29] Fung CSC, Yu EYT, Guo VY, Wong CKH, Kung K, Ho SY, Lam LY, Ip P, Fong DYT, Lam DCL. Development of a Health Empowerment Programme to improve the health of working poor families: protocol for a prospective cohort study in Hong Kong. BMJ Open. 2016;6: e010015. 26842271 10.1136/bmjopen-2015-010015 PMC4746471

[30] Hospital Authority: Hospital Authority Statistical Report (2011–2012). 2013. https://www.ha.org.hk/visitor/ha_visitor_index.asp?Content_ID=212922&Lang=ENG

[31] Two dishes and one soup" index rose 6%, Tung Chung is the most expensive https://ftu.org.hk/zh-hant/news/news_detail/?news_id=1220

[32] Ho KY, Li WH, Chung JOK, Lam KKW, Chan SS, Xia W. Factors contributing to the psychological well-being for Hong Kong Chinese children from low-income families: a qualitative study. Int J Ment Heal Syst. 2016;10:1–7. 10.1186/s13033-016-0088-0 PMC5016884 27617031

[33] Lam CLK, Guo VY, Wong CKH, Yu EYT, Fung CSC. Poverty and health-related quality of life of people living in Hong Kong: comparison of individuals from low-income families and the general population. J Public Health. 2017;39:258–65. 10.1093/pubmed/fdw046 27222238

[34] Tengland P-A. Empowerment: A conceptual discussion. Health Care Anal. 2008;16:77–96. 17985247 10.1007/s10728-007-0067-3

[35] Borges Rodrigues S, Parisod H, Barros L, Salanterä S. Examining empowerment interventions with families and preschool children: systematic review of randomized controlled trials. Health Educ Behav. 2022;49:358–77. 34515553 10.1177/10901981211031444

[36] Kwai Tsing Health City and Safe Community. https://www.cuhk.edu.hk/med/hep/research/pdf/reports/Kwai%20Tsing%202004.pdf

[37] Lai KY, Luk ES, Leung PW, Wong AS, Law L, Ho K. Validation of the Chinese version of the strengths and difficulties questionnaire in Hong Kong. Soc Psychiatry Psychiatr Epidemiol. 2010;45:1179–86. 19820885 10.1007/s00127-009-0152-z

[38] Strengths and Difficulties Questionnaire (SDQ) https://www.corc.uk.net/outcome-experience-measures/strengths-and-difficulties-questionnaire-sdq/

[39] Goodman A, Lamping DL, Ploubidis GB. When to use broader internalising and externalising subscales instead of the hypothesised five subscales on the Strengths and Difficulties Questionnaire (SDQ): data from British parents, teachers and children. J Abnorm Child Psychol. 2010;38:1179–91. 20623175 10.1007/s10802-010-9434-x

[40] Raat H, Botterweck AM, Landgraf JM, Hoogeveen WC, Essink-Bot M-L. Reliability and validity of the short form of the child health questionnaire for parents (CHQ-PF28) in large random school based and general population samples. J Epidemiol Community Health. 2005;59:75–82. 15598731 10.1136/jech.2003.012914 PMC1763365

[41] Child Health Questionnaire™ (CHQ™) https://www.qualitymetric.com/health-surveys/child-health-questionnaire-chq/

[42] Hsieh R, Hsueh Y, Huang H, Lin M, Lee W. The correlation of health of preschool Taiwanese children with developmental delay. HK J Paediatr (new series). 2013;18:159–66.

[43] Child health questionnaire (CHQ): Scoring and interpretation manual. https://www.healthactchq.com/survey/chq

[44] Landgraf J, Abetz L, Ware J: *Child Health Questionnaire (CHQ): a user’s manual.* Boston;; 1996.

[45] Wechsler D. Wechsler intelligence scale for children—4th edition (WISC-IV®). San Antonio, TX: The Psychological Corporation; 2003.

[46] Chan DW. Factor analysis of the HK-WISC at 11 age levels between 5 and 15 years. J Consult Clin Psychol. 1984;52:482.

[47] Lee L-MP, Lam YR: Confirmatory factor analyses of the Wechsler Intelligence Scale for Children-Revised and the Hong Kong-Wechsler Intelligence Scale for Children. *Educational and psychological measurement* 1988, 48:895–903.

[48] Median family income now $22k. https://www.news.gov.hk/en/categories/finance/html/2013/05/20130527_142244.shtml

[49] Lindacher V, Curbach J, Warrelmann B, Brandstetter S, Loss J. Evaluation of empowerment in health promotion interventions: a systematic review. Eval Health Prof. 2018;41:351–92. 29172696 10.1177/0163278716688065

[50] Boreham C, Riddoch C. The physical activity, fitness and health of children. J Sports Sci. 2001;19:915–29. 11820686 10.1080/026404101317108426

[51] Janssen I, LeBlanc AG. Systematic review of the health benefits of physical activity and fitness in school-aged children and youth. Int J Behav Nutr Phys Act. 2010;7:1–16. 20459784 10.1186/1479-5868-7-40 PMC2885312

[52] Hartzler AL, Tuzzio L, Hsu C, Wagner EH. Roles and functions of community health workers in primary care. The Annals of Family Medicine. 2018;16:240–5. 29760028 10.1370/afm.2208 PMC5951253

[53] Mensah FK, Kiernan KE. Parents’ mental health and children’s cognitive and social development: families in England in the Millennium Cohort Study. Soc Psychiatry Psychiatr Epidemiol. 2010;45:1023–35. 19823757 10.1007/s00127-009-0137-y

[54] Tahmassian K, Moghadam NJ. Relationship between self-efficacy and symptoms of anxiety, depression, worry and social avoidance in a normal sample of students. Iranian journal of psychiatry and behavioral sciences. 2011;5:91. 24644452 PMC3939966

[55] LCSD suspends enrolment arrangements of recreation and sports programmes. https://www.info.gov.hk/gia/general/202007/20/P2020072000474.htm

[56] Fröberg A, Jonsson L, Berg C, Lindgren E-C, Korp P, Lindwall M, Raustorp A, Larsson C. Effects of an Empowerment-Based Health-Promotion School Intervention on Physical Activity and Sedentary Time among Adolescents in a Multicultural Area. Int J Environ Res Public Health. 2018;15:2542. 30428548 10.3390/ijerph15112542 PMC6267499

[57] Lindgren E-C, Baigi A, Apitzsch E, Bergh H. Impact of a six-month empowerment-based exercise intervention programme in non-physically active adolescent Swedish girls. Health Educ J. 2011;70:9–20.

