# Supplementary material for: The 5-year outcomes of a health-empowerment program on low-income children’s behaviors and quality of life
Source: Child Adolesc Psychiatry Ment Health. 2024 Nov 10;18:144. doi: 10.1186/s13034-024-00834-9 (PMC11550554; doi:10.1186/s13034-024-00834-9)
Supplement: Supplementary file 1 — Additional file1 (DOCX 79 kb) [file 13034_2024_834_MOESM1_ESM.docx]

Supplementary Table 1. Main supportive services provided for TFES families

| Project Category | Project | Project Period |
| --- | --- | --- |
| Family / Community Harmony | Family College | 2012-Present |
| Family / Community Harmony | Parents Ambassador Mutual Support Programme | 2015-Present |
| Employment/Empowerment | Women Empowerment Project | 2012-2021 |
| Family / Community Harmony | Green Zero-dollar Shop | 2015-2021 |
| Education | Little Trekkers Learning Club | 2012-2022 |
| Education | Youth Development Project | 2018-2021 |
| Education | Green Trekkers | 2013-2017 |
| Health | Health Advice Station & Health Buddies | 2013-2017 |

Supplementary Table 2. Referral and counselling of participants with significant health risks

| Follow-up Management | Year | Number of Health Assessment participants | | Number referred (%) | |
| --- | --- | --- | --- | --- | --- |
|  |  | Children | Adults | Children | Adults |
| Referral to Optometrist | 2013-14 | -- | -- | -- | -- |
|  | 2014-15 | 211 | 193 | 81 (38.4%) | 68 (35.2%) |
|  | 2015-16 | 146 | 176 | 52 (35.6%) | 56 (31.8%) |
|  | 2016-17 | 167 | 183 | 34 (20.4%) | 25 (13.7%) |
|  | 2017-18 | 103 | 144 | 11 (10.7%) | 18 (12.5%) |
|  | 2018-19 | 118 | 198 | 5 (4.2%) | 17 (8.6%) |
|  | 2019-20 | 94 | 123 | 3 (3.2%) | 12 (9.8%) |
|  | 2020-21 | 41 | 84 | 3 (7.3%) | 14 (16.7%) |
|  | 2021-22 | 45 | 115 | 2 (4.4%) | 2 (1.8%) |
| Referral to Dentist | 2013-14 | -- | -- | -- | -- |
|  | 2014-15 | 211 | 193 | 59 (28.0%) | 97 (50.2%) |
|  | 2015-16 | 146 | 176 | 23 (15.8%) | 88 (50%) |
|  | 2016-17 | 167 | 183 | 23 (13.8%) | 65 (35.5%) |
|  | 2017-18 | 103 | 144 | 5 (4.9%) | 40 (27.8%) |
|  | 2018-19 | 118 | 198 | 13 (11.0%) | 67 (33.8%) |
|  | 2019-20 | 94 | 123 | 0 (0.0%) | 22 (17.9%) |
|  | 2020-21 | 41 | 84 | 0 (0.0%) | 32 (38.1%) |
|  | 2021-22 | 45 | 115 | 0 (0.0%) | 5 (4.4%) |
| Referral to General Out-Patient Clinic | 2013-14 | 489 | | 63 (25.8%) | |
|  | 2014-15 | 211 | 193 | 12 (5.7%) | 89 (46.1%) |
|  | 2015-16 | 146 | 176 | 3 (2.1%) | 38 (21.6%) |
|  | 2016-17 | 167 | 183 | 3 (1.8%) | 31 (16.9%) |
|  | 2017-18 | 103 | 144 | 2 (1.9%) | 39 (27.1%) |
|  | 2018-19 | 118 | 198 | 1 (0.8%) | 37 (18.7%) |
|  | 2019-20 | 94 | 123 | 1 (1.1%) | 29 (23.6%) |
|  | 2020-21 | 41 | 84 | 0 (0.0%) | 34 (40.5%) |
|  | 2021-22 | 45 | 115 | 2 (4.4%) | 25 (21.7%) |
| Referral to Specialist Outpatient Clinic | 2013-14 | 489 | | 12 (4.9%) | |
|  | 2014-15 | 211 | 193 | 9 (4.3%) | 7 (3.6%) |
|  | 2015-16 | 146 | 176 | 1 (0.7%) | 18 (10.2%) |
|  | 2016-17 | 167 | 183 | 0 (0.0%) | 16 (8.7%) |
|  | 2017-18 | 103 | 144 | 1 (1.0%) | 19 (13.2%) |
|  | 2018-19 | 118 | 198 | 0 (0.0%) | 13 (6.6%) |
|  | 2019-20 | 94 | 123 | 1 (1.1%) | 12 (9.8%) |
|  | 2020-21 | 41 | 84 | 0 (0.0%) | 8 (9.5%) |
|  | 2021-22 | 45 | 115 | 1 (2.2%) | 9 (7.9%) |
| Referral to Social Workers | 2013-14 | 489 | | 5 (2%) | |
|  | 2014-15 | 211 | 193 | 8 (3.8%) | 8 (4.1%) |
|  | 2015-16 | 146 | 176 | 0 (0.0%) | 3 (1.7%) |
|  | 2016-17 | 167 | 183 | 9 (5.4%) | 6 (3.3%) |
|  | 2017-18 | 103 | 144 | 1 (1.0%) | 1 (0.7%) |
|  | 2018-19 | 118 | 198 | 3 (2.5%) | 2 (1.0%) |
|  | 2019-20 | 94 | 123 | 0 (0.0%) | 3 (2.4%) |
|  | 2020-21 | 41 | 84 | 0 (0.0%) | 2 (2.4%) |
|  | 2021-22 | 45 | 115 | 1 (2.2%) | 0 (0.0%) |
| Referral to Child Assessment Centre | 2013-14 | 245 | -- | 2 (0.8%) | -- |
|  | 2014-15 | 211 | -- | 10 (4.7%) | -- |
|  | 2015-16 | 146 | -- | 1 (0.7%) | -- |
|  | 2016-17 | 167 | -- | 0 (0.0%) | -- |
|  | 2017-18 | 103 | -- | 0 (0.0%) | -- |
|  | 2018-19 | 118 | -- | 0 (0.0%) | -- |
|  | 2019-20 | 94 | -- | 0 (0.0%) | -- |
|  | 2020-21 | 41 | -- | 0 (0.0%) | -- |
|  | 2021-22 | 45 | -- | 1 (2.2%) | -- |

Note: Total % may not add up to 100% because of rounding.

Supplementary Table 3. Health enablement activities

|  | Date | Content | Number of participants |
| --- | --- | --- | --- |
| 2013-2014 | 10th Apr, 17th Apr, 24th Apr, 8th May, 15th May, 22nd May (6 sessions) | Mental health enhancement | 9 |
| 2014-2015 | 26th Apr, 3rd May, 11th May, 17th May, 25th May, 31st May, 8th Jun, 14th Jun (8 sessions) | Eight-sectioned exercise 1 | 54 |
|  | 19th Jul, 26th Jul, 9th Aug, 16th Aug, 23rd Aug, 30th Aug, 6th Sep, 13th Sep (8 sessions) | Eight-sectioned exercise 2 |  |
|  | 1st Nov, 8th Nov, 22nd Nov, 29th Nov, 6th Dec, 20th Dec (6 sessions) | Tai Chi |  |
|  | January to July in 2014 (15 sessions) | Family well-being enablement counselling | 15 |
|  | May and August 2014 (2 sessions) | Family well-being workshops | 5 |
| 2015-2016 | 30th May, 6th Jun, 13th Jun, 20th Jun, 27th Jun, 4th Jul, 11th Jul, 18th Jul (8 sessions) | Eight-sectioned exercise | 35 |
|  | 28th Mar, 4th Apr, 11th Apr, 18th Apr, 25th Apr, 2nd May, 9th May, 16th May (8 sessions) | Mawangdui Daoyin Exercises | 35 |
|  | 10th Oct, 17th Oct, 24th Oct, 31st Oct, 7th Nov, 21st Nov, 28th Nov 2015, 16th Jan 2016 (8 sessions) | Physiotherapy class | 50 |
| 2016-2017 | 12 Mar (1 sessions) | Nutrition education for children | 27 |
|  | 9th Apr, 16th Apr, 23rd Apr, 30th Apr (4 sessions) | Nutrition Workshop | 31 |
|  | 16th Jul, 23th Jul, 30th Jul, 6th Aug (4 sessions) | Kids and parents learn healthy receipts together | 27 |
|  | 9th Jul, 16th Jul, 23rd Jul, 30th Jul (4 sessions) | Boxing Class for teenagers | 14 |
|  | 4th Aug, 11th Aug, 18th Aug, 30th Aug (4 sessions) | Dancing Class for teenagers | 11 |
|  | 8th Oct, 15th Oct, 22th Oct, 29th Oct, 5th Nov, 12th Nov, 19th Nov, 3rd Dec (8 sessions) | Parents exercise together with their children | 27 |
| 2017-2018 | 22ed Apr, 29th Apr, 6th May, 13th May (4 sessions) | Kids and parents learn healthy receipts together | 30 |
|  | 3rd Mar, 10th Mar, 17th Mar, 24th Mar (4 sessions) | Rope Skipping Class 1 | 34 |
|  | 3rd Aug, 4th Aug, 8th Aug, 10th Aug (4 sessions) | Rope Skipping Class 2 |  |
|  | 11st Nov, 25th Nov, 9th Dec, 23rd Dec (4 sessions) | Rope Skipping Class 3 |  |
|  | 14th Sep, 21st Sep, 23rd Sep, 28th Sep, 12ed Oct, 21st Oct (6 sessions) | Dancing Gym | 25 |
| 2018-2019 | 5th May, 15th May, 26th May, 2nd Jun (4 sessions) | Kids and parents learn healthy receipts together | 19 |
|  | 25th Apr, 2nd May, 16th May, 30th May, 6th Jun, 12th Sep (6 sessions) | Dancing Gym 1 | 38 |
|  | 19th Sep, 26th Sep, 3rd Oct, 10th Oct, 23rd Oct, 2018 (6 sessions) | Dancing Gym 2 |  |
|  | 20th Feb, 27th Feb, 6th Mar, 13th Mar, 20th Mar, 27th Mar (6 sessions) | Dancing Gym 3 |  |
|  | 19th Jul, 7th Aug, 13th Aug, 14th Aug and 16th Aug (5 sessions) | Rope Skipping Class 1 | 20 |
|  | 10th Nov, 17th Nov, 8th Dec, 15th Dec, 2018, 5th Jan, 26th Jan, 2019 (6 sessions) | Rope Skipping Class 2 |  |
| 2019-2020 | 4th May, 11th May, 18th May, 25th May, 2019 (4 sessions) | Kids and parents learn healthy receipts together | 17 |
|  | 29th May, 5th Jun, 12th Jun, 19th Jun, 26th Jun, 3rd Jul (6 sessions) | Dancing Gym 1 | 34 |
|  | 11th Sep, 18th Sep, 25th Sep, 2nd Oct, 9th Oct, 16th Oct, 2019 (6 sessions) | Dancing Gym 2 |  |
|  | 30th Jul (1 sessions) | Summer sports | 10 |
|  | 2-Nov, 9-Nov, 23-Nov, 30-Nov-19 (4 sessions) | Walking group | 20 |
| 2020-2021 | 1st Aug, 8th Aug, 15th Aug, 22nd Aug (4 sessions) (Via ZOOM) | Kids and parents learn healthy receipts together | 12 |
|  | 27th Aug, 3rdth Aug, 10th Aug, 17th Aug, 24th Aug, 31st Aug (6 sessions) (Via ZOOM) | Dancing Gym 1 | 27 |
|  | 16th Dec, 23rd Dec, 30th Dec, 2020, 6th Jan, 13th Jan, 20 Jan, 24th Jan, 3rd (8 sessions) (Via ZOOM) | Dancing Gym 2 |  |
|  | Mar, 10th Mar, 17th Mar, 24th Mar, 31st Mar, 2021 (5 sessions) (Via ZOOM) | Dancing Gym 3 |  |
| 2021-2022 | 24-Jul, 31-Jul, 7-Aug, 14-Aug-21 (4 sessions) (Via ZOOM) | Healthy Home Cooking  Workshops | 12 |
|  | 26-May, 2-Jun, 9-Jun, 16-Jun, 23-Jun, 30-Jun-21 (4 sessions) (Via ZOOM) | Dancing Gym | 14 |
|  | 12-Nov-21, 20-Nov, 27-Nov, 4-Dec-21 (4 sessions) | Walking Group 2021 | 15 |

Supplementary Table 4. Health talks and seminars

|  | Date | Content | Number of participants |
| --- | --- | --- | --- |
| 2013-2014 | 30 Nov 2013 | Oral health, oral hygiene practice and common oral/dental problem, Weight management, healthy cooking technique and food selection | 10 |
| 2014-2015 | 2nd Mar 2014 | Qigong | 20 |
|  | 24th May 2014 | Ergonomics and Exercises & Fitness Training Help to Protect from Back Pain | 19 |
|  | 5th Jul 2014 | Liver Disease Management- Liver Disease | 11 |
|  | 13th Dec 2014 | Body Constitutional Types in Chinese Medicine | 7 |
| 2015-2016 | 11th Apr 2015 | Common Eating Problems | 26 |
|  | 9th May 2015 | Introduce My Plate |  |
|  | 13th Jun 2015 | Fat Facts: Know More about Oil |  |
|  | 18th Jul 2015 | Summary and Nutrition Q & A |  |
|  | 5th Dec 2015 | Body Constitutional Types in Chinese Medicine | 44 |
|  | 5th Dec 2015 | Common Female Health Problems |  |
| 2016-2017 | 9th Apr 2016 | Knowing My plate | 31 |
|  | 16th Apr 2016 | Tasting Event– Try new foods |  |
|  | 23rd Apr 2016 | Supermarket Field Trip – label reading |  |
|  | 30th Apr 2016 | Family Cooking |  |
|  | 17th De 2016 | Men’s Body Constitutional Types in Chinese Medicine | 11 |
|  | 17th Dec 2016 | Men’s Health Problems |  |
| 2017-2018 | 22nd Apr 2017 | Knowing your nutrition need | 30 |
|  | 29th Apr 2017 | How to value food |  |
|  | 6th May 2017 | Recipe revamp |  |
|  | 13th May 2017 | Be a smart eater |  |
|  | 4 Nov 2017 | Sexual Health of Teenager | 14 |
|  | 4 Nov 2017 | Positive Psychology |  |
| 2018-2019 | 5th May 2018 | Knowing My Signature Dish | 19 |
|  | 15th May 2018 | Turning old to new |  |
|  | 26th May 2018 | Adding color to your dish |  |
|  | 2nd Jun 2018 | Menu of the Week |  |
|  | 1st Dec 2018 | Understand the causes, screening for H. Pylori, other clinical assessments, management by western medication- from the view of Western Medicine | 12 |
|  | 1st Dec 2018 | Understand the causes, management for different body constitutions- from the view of Chinese Medicine. |  |
| 2019-2020 | 4th May 2019 | Healthy Home Cooking -Foods for chronic disease prevention | 17 |
|  | 11th May 2019 | Healthy Home Cooking -Recommendations for festive dishes |  |
|  | 18th May 2019 | Healthy Home Cooking -Food recommendations for children and older adults |  |
|  | 25th May 2019 | Healthy Home Cooking -Foods for skin care and improving immune system |  |
|  | 30th Aug 2019 | Nutrition Talk –Understanding the concept of “fruits and vegetables 2+3 a day” and tips for practice | 9 |
|  | 25th Oct 2019 | Dental Talk –Focus on understanding dental problems and methods of prevention | 22 |
| 2020-2021 | 1st Aug 2020 | Healthy Home Cooking - Introduction to the concept of the ‘three LOWs’ | 12 |
|  | 8th Aug 2020 | Healthy Home Cooking - Sodium in food/condiments and use of low sodium alternatives in cooking |  |
|  | 15th Aug 2020 | Healthy Home Cooking - Identification of healthier cooking methods and ingredients |  |
|  | 22nd Aug 2020 | Healthy Home Cooking - Recognizing hidden sugar in food and know how to cut down on sugar intake or find alternatives when craving |  |
|  | 9th Jan 2021 | Stress Management Talk (Via ZOOM) | 25 |
|  | 20th Feb 2021 | Visual Health Talk (Via ZOOM) | 24 |
| 2021-2022 | 12^th^ March 2022 | Seminar on “Reversing Pre-DM”: Introduction to prediabetes and the linkage between prediabetes and diabetes; suggest evidence-based strategies to prevent diabetes (Via ZOOM) | 25 |
|  | 24^th^ Jul 2021 | Healthy Home Cooking Workshops: Immune System –  Introduction to the immune system and food that enhance and reduce immunity. (Via ZOOM) | 12 |
|  | 31^st^ Jul 2021 | Healthy Home Cooking Workshops: Gut – Introduce  diseases related to gut health and food that enhance and reduce gut health. (Via ZOOM) |  |
|  | 7^th^ Aug 2021 | Healthy Home Cooking Workshops: Introduce  stress management tips and diets that help to relieve stress. (Via ZOOM) |  |
|  | 14^th^ Aug 2021 | Healthy Home Cooking Workshops: Recent Diet Trends –  Explain the mechanism behind Keto and fasting while re-evaluating their effectiveness on weight control. (Via ZOOM) |  |

Supplementary Table 5. Baseline characteristics of Mother.

| **Mother’s socioeconomic status** | | | | |
| --- | --- | --- | --- | --- |
|  | Total | Intervention | Comparison | *p-value* |
| **Age, years** | *N*=222 | *N*=115 | *N*=107 | 0.104 |
|  | 39.63±5.51 | 39.05±5.14 | 40.25±5.85 |  |
|  |  |  |  |  |
| **Body weight status** | *N*=235 | *N*=115 | *N*=107 | 0.412 |
| Normal weight | 144(64.86%) | 75(65.22%) | 69(64.49%) |  |
| Overweight | 63(28.38%) | 30(26.09%) | 33(30.84%) |  |
| obese | 15(6.76%) | 10(8.70%) | 5(4.67%) |  |
|  |  |  |  |  |
| **Chronic disease** | *N* =222 | *N* =115 | *N* =107 | 0.569 |
| Yes | 87(39.19%) | 43(37.39%) | 44(41.12%) |  |
| No | 135(60.81%) | 72(62.61%) | 63(58.88%) |  |
|  |  |  |  |  |
| **Educational level** | *N* =226 | *N* =119 | *N* =107 | 0.668 |
| No/primary education | 33(14.60%) | 19(15.97%) | 14(13.08%) |  |
| Secondary education | 175(77.43%) | 92(77.31%) | 83(77.57%) |  |
| Higher education | 18(7.96%) | 8(6.72%) | 10(9.35%) |  |
|  |  |  |  |  |
| **Working status** | *N* =226 | *N* =119 | *N* =107 | 0.001* |
| Current working | 83(36.73%) | 57 (47.90%) | 26(24.30%) |  |
| Homeworker | 138(61.06%) | 59(49.58%) | 79(73.83%) |  |
| Not working | 5(2.21%) | 3(2.52%) | 2(1.87%) |  |

Supplementary Table 6. Changes in individual domains within CHQ over 5-year follow-up

| **CHQ domains** | Baseline |  | Follow-up |  | 5-year change (Follow-up – Baseline) in score | | |
| --- | --- | --- | --- | --- | --- | --- | --- |
|  | Mean±SD |  | Mean±SD |  | Mean±SD | Cohen’s *d* | *p*-value |
| GH | 62.16 ± 20.25 |  | 63.79 ± 20.15 |  | 1.86 ± 23.06 | 0.08 | 0.223 |
| PF | 91.25 ± 17.53 |  | 92.89 ± 18.57 |  | 1.29 ± 22.61 | 0.09 | 0.389 |
| REP | 78.80 ± 29.42 |  | 89.40 ± 22.02 |  | 10.82 ± 31.60 | 0.41 | <0.001* |
| RP | 90.90 ± 19.96 |  | 94.28 ± 15.27 |  | 3.38 ± 23.54 | 0.19 | 0.032* |
| BP | 87.72 ± 17.41 |  | 90.54 ± 14.29 |  | 2.98 ± 17.66 | 0.18 | 0.011* |
| BE | 66.11 ± 20.67 |  | 72.56 ± 19.91 |  | 6.70 ± 20.97 | 0.32 | <0.001* |
| MH | 81.20 ± 20.74 |  | 86.02 ± 18.70 |  | 4.40 ± 21.67 | 0.24 | 0.002* |
| SE | 64.97 ± 13.96 |  | 68.32 ± 14.22 |  | 3.47 ± 16.00 | 0.24 | 0.001* |
| PE | 75.77 ± 21.78 |  | 84.36 ± 18.03 |  | 9.07 ± 21.91 | 0.43 | <0.001* |
| PT | 78.56 ± 23.94 |  | 85.98 ± 21.98 |  | 7.34 ± 24.29 | 0.32 | <0.001* |
| FA | 81.53 ± 23.63 |  | 90.90 ± 16.15 |  | 9.46 ± 24.43 | 0.47 | <0.001* |
| FC | 60.33 ± 25.64 |  | 60.42 ± 26.69 |  | -0.09 ± 29.79 | 0.00 | 0.965 |

- Emotional/Behavioral; RP = Role/Social Limitations – Physical; BP = Bodily Pain/Discomfort; BE = Behavior; MH = Mental Health; SE = Self-Esteem; PE = Parent Impact – Emotion; PT = Parent Impact – Time; FA = Family Activities; FC = Family Cohesion.

Notes: **p* value by paired sample t test; Cohen’s effect size d = difference between baseline and follow-up scores/ pooled standard deviations of the two scores

Supplementary Table 7. Comparison of Changes in individual domains within CHQ between Intervention groups and Comparison groups.

|  | Baseline | | Follow-up | | 5-year change (Follow-up – Baseline) in score | | | | | |
| --- | --- | --- | --- | --- | --- | --- | --- | --- | --- | --- |
| **CHQ domains** | Intervention group | Comparison group | Intervention group | Comparison group | Intervention group  (Follow-up period ranged from 42 to 96 months) | | | Comparison group  (Follow-up period ranged from 42 to 92 months) | | |
|  | *N* = 124 | *N* = 115 | *N* = 124 | *N* = 115 | *N* = 124 | | | *N* = 115 | | |
|  | Mean±SD | Mean±SD | Mean±SD | Mean±SD | Mean±SD | Cohen’s *d* | *p*-value | Mean±SD | Cohen’s *d* | *p*-value |
| GH | 62.27±19.98 | 62.05±20.60 | 64.12±21.54 | 63.42±18.62 | 2.30±23.88 | 0.09 | 0.305 | 1.43±22.30 | 0.07 | 0.496 |
| PF | 92.35 ±16.37 | 90.16±18.63 | 95.70±13.19 | 89.86±22.68 | 2.97±20.67 | 0.23 | 0.128 | -0.39±24.38 | -0.01 | 0.865 |
| REP | 80.12±29.32 | 77.49±29.58 | 91.40±18.95 | 87.25±24.81 | 11.99±31.40 | 0.46 | <0.001* | 9.65±31.89 | 0.36 | 0.002* |
| RP | 90.56±20.64 | 91.23±19.36 | 94.35±14.55 | 94.20±16.08 | 3.83±25.10 | 0.21 | 0.107 | 2.92±21.98 | 0.17 | 0.158 |
| BP | 90.00±14.87 | 85.44±19.42 | 91.13±12.04 | 89.91±16.41 | 1.58±16.65 | 0.08 | 0.313 | 4.39±18.58 | 0.25 | 0.013* |
| BE | 68.18±22.07 | 64.04±19.05 | 76.01±19.25 | 68.85±20.01 | 8.60±21.47 | 0.38 | <0.001* | 4.80±20.38 | 0.25 | 0.013* |
| MH | 82.09±20.90 | 80.30±20.64 | 88.91±16.93 | 82.90±10.05 | 6.21±21.94 | 0.36 | 0.003* | 2.60±21.34 | 0.13 | 0.197 |
| SE | 66.19±14.75 | 63.74±13.07 | 68.95±14.47 | 67.64±13.98 | 2.89±16.90 | 0.19 | 0.071 | 4.06±15.10 | 0.29 | 0.005* |
| PE | 76.23±22.70 | 75.33±20.93 | 83.47±20.26 | 85.33±15.29 | 8.04±22.94 | 0.34 | <0.001* | 10.09±20.90 | 0.55 | <0.001* |
| PT | 80.09±24.18 | 77.05±23.71 | 90.05±18.40 | 81.59±24.62 | 10.03±24.35 | 0.47 | <0.001* | 4.68±24.04 | 0.19 | 0.040* |
| FA | 80.97±25.83 | 82.08±21.32 | 91.53±16.38 | 90.22±15.95 | 10.95±26.21 | 0.49 | <0.001* | 7.96±22.54 | 0.43 | <0.001* |
| FC | 63.41±25.05 | 57.28±25.96 | 63.63±26.64 | 56.96±26.42 | 0.40±30.82 | 0.01 | 0.891 | -0.57±28.87 | -0.12 | 0.833 |

CHQ = Child Health Questionnaire Parent Form 28;GH= General Health; PF = Physical Functioning; REB = Role/Social Limitations - Emotional/Behavioral; RP = Role/Social Limitations – Physical; BP = Bodily Pain/Discomfort; BE = Behavior; MH = Mental Health; SE = Self-Esteem; PE = Parent Impact – Emotion; PT = Parent Impact – Time; FA = Family Activities; FC = Family Cohesion.

Notes: **p* value by paired sample t test; Cohen’s effect size d = difference between baseline and follow-up scores/ pooled standard deviations of the two scores

Supplementary Table 8. Association between Health Empowerment Program and changes in individual domains within CHQ after 5-year follow-up

| **CHQ domains** | B (95% CI) | *p*-value for *β* | *Adjusted R^2^* | *F-ratio* | *P value for F ratio* |
| --- | --- | --- | --- | --- | --- |
| GH | 1.74( -3.61, 7.09) | 0.522 | 0.29 | 7.44 | <0.001* |
| PF | 5.42(0.23, 10.60) | 0.041* | 0.33 | 8.64 | <0.001* |
| REP | 4.59( -1.16, 10.35) | 0.117 | 0.57 | 21.29 | <0.001* |
| RP | -0.06( -4.28, 4.16) | 0.977 | 0.59 | 23.42 | <0.001* |
| BP | 0.26(-3.45, 3.97) | 0.890 | 0.44 | 13.16 | <0.001* |
| BE | 7.44(2.67, 12.22) | 0.002* | 0.33 | 8.71 | <0.001* |
| MH | 4.67(-0.13,9.47) | 0.056 | 0.37 | 9.95 | <0.001* |
| SE | 0.75(-2.99,4.48) | 0.695 | 0.29 | 7.42 | <0.001* |
| PE | -1.74(-6.16,2.68) | 0.438 | 0.49 | 15.66 | <0.001* |
| PT | 7.22(1.91,12.53) | 0.008* | 0.40 | 11.39 | <0.001* |
| FA | 2.03( -2.26, 6.32) | 0.353 | 0.60 | 24.06 | <0.001* |
| FC | 5.72(-1.21, 12.64) | 0.105 | 0.30 | 7.68 | <0.001* |

CHQ = Child Health Questionnaire Parent Form 28;GH= General Health; PF = Physical Functioning; REB = Role/Social Limitations - Emotional/Behavioral; RP = Role/Social Limitations – Physical; BP = Bodily Pain/Discomfort; BE = Behavior; MH = Mental Health; SE = Self-Esteem; PE = Parent Impact – Emotion; PT = Parent Impact – Time; FA = Family Activities; FC = Family Cohesion.

Notes: Children’ age, gender, IQ level, learning disability, body weight status, chronic disease history, scores on baseline and parents’ marital status, family monthly income, government CSSA status were adjusted for in the multiple linear regressions. **p* value <0.05 is statistically significant

Supplementary Table 9. Subgroup analysis -association between Health Empowerment Program and changes in outcomes after 5-year follow-up

|  | B (95% CI) | *p*-value for *β* | *R^2^* | *Adjusted R^2^* | *F* | *p > F* | *P for interaction* |
| --- | --- | --- | --- | --- | --- | --- | --- |
| **SDQ** |  |  |  |  |  |  |  |
| **Emotional symptoms** | | | | | | | |
| Age, years |  |  |  |  |  |  |  |
| 6-7 | 0.05( -1.16, 1.27) | 0.928 | 0.41 | 0.23 | 2.33 | 0.022 | 0.884 |
| 8-11 | -0.11(-0.60, 0.38) | 0.656 | 0.54 | 0.50 | 14.17 | <0.001* |  |
| Gender |  |  |  |  |  |  |  |
| Male | -0.07(-0.72, 0.58) | 0.840 | 0.42 | 0.35 | 5.77 | <0.001* | 0.596 |
| Female | -0.21(-0.83,0.40) | 0.488 | 0.62 | 0.57 | 12.27 | <0.001* |  |
| Chronic diseases |  |  |  |  |  |  |  |
| Yes | 0.24(-1.27, 1.74) | 0.748 | 0.42 | 0.16 | 1.63 | 0.151 | 0.505 |
| No | -0.19(-0.65, 0.26) | 0.402 | 0.54 | 0.50 | 15.75 | <0.001* |  |
| Government CSSA |  |  |  |  |  |  |  |
| Yes | -0.43(-1.59,0.74) | 0.465 | 0.53 | 0.37 | 3.35 | 0.002* | 0.787 |
| No | -0.06(-0.54,0.42) | 0.807 | 0.54 | 0.50 | 14.37 | <0.001* |  |
| **Conduct problems** | | | | | | | |
| Age, years |  |  |  |  |  |  |  |
| 6-7 | -1.15(-2.06, -0.25) | 0.014* | 0.65 | 0.55 | 6.44 | <0.001* | 0.309 |
| 8-11 | -0.62(-1.03, -0.22) | 0.003* | 0.54 | 0.51 | 14.53 | <0.001* |  |
| Gender |  |  |  |  |  |  |  |
| Male | -0.64(-1.19, -0.85) | 0.024* | 0.56 | 0.50 | 9.79 | <0.001* | 0.809 |
| Female | 0.09(-0.81,0.99) | 0.842 | 0.17 | 0.06 | 1.52 | 0.123 |  |
| Chronic diseases |  |  |  |  |  |  |  |
| Yes | -0.05(-1.83,1.74) | 0.958 | 0.24 | -0.1 | 0.71 | 0.714 | 0.256 |
| No | -1.0(-1.5, -0.46) | <0.001* | 0.17 | 0.11 | 2.82 | 0.001 |  |
| Government CSSA |  |  |  |  |  |  |  |
| Yes | -0.62(-1.48,0.23) | 0.149 | 0.67 | 0.56 | 6.04 | <0.001* | 0.303 |
| No | -0.68(-1.08, -0.27) | 0.001* | 0.55 | 0.51 | 15.09 | <0.001* |  |
| **Hyperactivity inattention** | | | | | | | |
| Age, years |  |  |  |  |  |  |  |
| 6-7 | -1.48(-2.81, -0.14) | 0.031* | 0.44 | 0.28 | 2.68 | 0.009* | 0.307 |
| 8-11 | -0.67(-1.18, -0.15) | 0.011* | 0.34 | 0.34 | 0.28 | <0.001* |  |
| Gender |  |  |  |  |  |  |  |
| Male | -0.74(-1.45, -0.03) | 0.041* | 0.36 | 0.28 | 4.46 | <0.001* | 0.803 |
| Female | -0.83(-1.51, -0.14) | 0.018* | 0.35 | 0.26 | 3.96 | <0.001* |  |
| Chronic diseases |  |  |  |  |  |  |  |
| Yes | -0.93(-2.81, 0.95) | 0.319 | 0.34 | 0.05 | 1.17 | 0.357 | 0.574 |
| No | -0.61(-1.1, -0.12) | 0.016* | 0.33 | 0.28 | 6.58 | <0.001* |  |
| Government CSSA |  |  |  |  |  |  |  |
| Yes | -0.59(-1.69, 0.50) | 0.279 | 0.47 | 0.29 | 2.66 | 0.009* | 0.283 |
| No | -0.77(-1.3, -0.24) | 0.005* | 0.37 | 0.31 | 7.07 | <0.001* |  |
| **Peer problem** | | | | | | | |
| Age, years |  |  |  |  |  |  |  |
| 6-7 | -1.00(-2.02, 0.01) | 0.053 | 0.75 | 0.68 | 10.52 | <0.001* | 0.005* |
| 8-11 | 0.00(-0.46,0.47) | 0.983 | 0.50 | 0.46 | 12.20 | <0.001* |  |
| Gender |  |  |  |  |  |  |  |
| Male | -0.28(-0.97,0.42) | 0.435 | 0.49 | 0.42 | 7.42 | <0.001* | 0.943 |
| Female | -0.22(-0.77,0.33) | 0.428 | 0.60 | 0.55 | 11.19 | <0.001* |  |
| Chronic diseases |  |  |  |  |  |  |  |
| Yes | 0.72(-0.30,1.74) | 0.158 | 0.68 | 0.54 | 4.77 | 0.001* | 0.011* |
| No | -0.60(-1.08, -0.11) | 0.016* | 0.50 | 0.47 | 13.70 | <0.001* |  |
| Government CSSA |  |  |  |  |  |  |  |
| Yes | -0.19(-1.54,1.17) | 0.784 | 0.49 | 0.32 | 2.91 | 0.005* | 0.587 |
| No | -0.40(-0.85, 0.05) | 0.084 | 0.56 | 0.52 | 15.55 | <0.001* |  |
| **Prosocial behavior** | | | | | | | |
| Age, years |  |  |  |  |  |  |  |
| 6-7 | 1.65(0.42, 2.88) | 0.010* | 0.42 | 0.25 | 2.45 | 0.017* | 0.026* |
| 8-11 | 0.19(-0.40, 0.78) | 0.527 | 0.43 | 0.38 | 9.11 | <0.001* |  |
| Gender |  |  |  |  |  |  |  |
| Male | 0.44(-0.29, 1.17) | 0.235 | 0.45 | 0.38 | 6.48 | <0.001* | 0.433 |
| Female | 0.67(-0.09, 1.43) | 0.084 | 0.37 | 0.28 | 4.31 | <0.001* |  |
| Chronic diseases |  |  |  |  |  |  |  |
| Yes | 0.18(-1.02, 1.38) | 0.756 | 0.59 | 0.41 | 3.29 | 0.007* | 0.990 |
| No | 0.56(-0.01, 1.14) | 0.054 | 0.37 | 0.32 | 7.95 | <0.001* |  |
| Government CSSA |  |  |  |  |  |  |  |
| Yes | 0.43(-0.80, 1.66) | 0.484 | 0.38 | 0.18 | 1.86 | 0.067 | 0.745 |
| No | 0.51(-0.09, 1.10) | 0.094 | 0.40 | 0.35 | 8.06 | <0.001* |  |
| **Total difficulties** | | | | | | | |
| Age, years |  |  |  |  |  |  |  |
| 6-7 | -4.04(-7.15, -0.93) | 0.012* | 0.57 | 0.45 | 4.58 | <0.001* | 0.055 |
| 8-11 | -1.04(-2.78, -0.04) | 0.044* | 0.37 | 0.32 | 7.20 | <0.001* |  |
| Gender |  |  |  |  |  |  |  |
| Male | -1.92(-3.85, 0.01) | 0.051 | 0.37 | 0.29 | 4.56 | <0.001* | 0.842 |
| Female | -1.88(-3.51, -0.25) | 0.024* | 0.48 | 0.42 | 7.00 | <0.001* |  |
| Chronic diseases |  |  |  |  |  |  |  |
| Yes | -0.41(-4.10, 3.28) | 0.822 | 0.45 | 0.21 | 1.89 | 0.091 | 0.284 |
| No | -2.19(-3.53, -0.84) | 0.002* | 0.39 | 0.35 | 8.75 | <0.001* |  |
| Government CSSA |  |  |  |  |  |  |  |
| Yes | -1.87(-5.07, 1.32) | 0.243 | 0.51 | 0.34 | 3.10 | 0.003* | 0.306 |
| No | -2.03(-3.38, -0.68) | 0.003* | 0.42 | 0.37 | 8.84 | <0.001* |  |
| **CHQ** | | | | | | | |
| **PHS** | | | | | | | |
| Age, years |  |  |  |  |  |  |  |
| 6-7 | 1.92(-2.36, 6.20) | 0.369 | 0.57 | 0.43 | 4.22 | <0.001* | 0.654 |
| 8-11 | 0.53(-2.15, 3.21) | 0.696 | 0.37 | 0.32 | 6.85 | <0.001* |  |
| Gender |  |  |  |  |  |  |  |
| Male | 3.60(0.23, 6.97) | 0.037* | 0.45 | 0.37 | 5.69 | <0.001* | 0.024* |
| Female | -1.45(-4.38, 1.48) | 0.329 | 0.44 | 0.37 | 5.88 | <0.001* |  |
| Chronic diseases |  |  |  |  |  |  |  |
| Yes | 4.31(-2.04, 10.66) | 0.174 | 0.52 | 0.32 | 2.57 | 0.023* | 0.378 |
| No | 0.22(-2.15, 2.61) | 0.852 | 0.39 | 0.34 | 7.88 | <0.001* |  |
| Government CSSA |  |  |  |  |  |  |  |
| Yes | 6.44(-0.09, 12.97) | 0.053 | 0.46 | 0.26 | 2.33 | 0.023* | 0.423 |
| No | 0.02(-2.31, 2.35) | 0.989 | 0.42 | 0.37 | 8.56 | <0.001* |  |
| **PSS** | | | | | | | |
| Age, years |  |  |  |  |  |  |  |
| 6-7 | 7.04(0.19, 13.89) | 0.044* | 0.46 | 0.30 | 2.79 | 0.008* | 0.134 |
| 8-11 | 2.13(-0.36, 4.63) | 0.093 | 0.49 | 0.45 | 11.22 | <0.001* |  |
| Gender |  |  |  |  |  |  |  |
| Male | 1.85(-1.84, 5.54) | 0.323 | 0.39 | 0.31 | 4.54 | <0.001* | 0.290 |
| Female | 4.01(0.91, 7.12) | 0.012* | 0.55 | 0.49 | 9.01 | <0.001* |  |
| Chronic diseases |  |  |  |  |  |  |  |
| Yes | 0.24(-8.12, 8.60) | 0.954 | 0.39 | 0.14 | 1.53 | 0.180 | 0.211 |
| No | 3.45(1.01, 5.90) | 0.006* | 0.48 | 0.44 | 11.76 | <0.001* |  |
| Government CSSA |  |  |  |  |  |  |  |
| Yes | 5.32(-1.69, 12.34) | 0.133 | 0.47 | 0.28 | 2.47 | 0.016* | 0.741 |
| No | 2.45(-0.08, 4.97) | 0.057 | 0.47 | 0.42 | 10.25 | <0.001* |  |

SDQ= Strengths and Difficulties Questionnaire; CHQ = Child Health Questionnaire Parent Form 28; PHS = Physical Summary score; PSS = Psychosocial Summary score

Notes: Children’ age, gender, IQ level, learning disability, body weight status, chronic disease history, scores on baseline and parents’ marital status, family monthly income, government CSSA status were adjusted for in the multiple linear regressions. **p* value <0.05 is statistically significant

Supplementary Table 10. Sensitivity analysis of the association between Health Empowerment Program and change in outcomes after 5-year follow-up, with additional adjustment for the mothers’ sociodemographic and health status

|  | B (95% CI) | *p*-value for *β* | *Adjusted R^2^* | *F ratio* | *P value for F ratio* |
| --- | --- | --- | --- | --- | --- |
| **SDQ** |  |  |  |  |  |
| Emotional symptoms | -0.18( -0.64, 0.29) | 0.454 | 0.49 | 10.33 | <0.001* |
| Conduct problems | -0.72( -1.11, -0.34) | <.001* | 0.53 | 12.15 | <0.001* |
| Hyperactivity inattention | -0.77( -1.28, -0.26) | 0.003* | 0.28 | 4.86 | <0.001* |
| Peer problem | -0.44( -0.92, 0.04) | 0.071 | 0.45 | 9.10 | <0.001* |
| Prosocial behavior | 0.57(0.03, 1.12) | 0.040* | 0.32 | 5.66 | <0.001* |
| Total difficulties | -2.19( -3.50, -0.88) | 0.001* | 0.38 | 6.94 | <0.001* |
| **CHQ** |  |  |  |  |  |
| PHS | -0.15( -2.53, 2.24) | 0.903 | 0.35 | 5.95 | <0.001* |
| PSS | 3.67(1.22, 6.12) | 0.003* | 0.40 | 7.12 | <0.001* |

SDQ= Strengths and Difficulties Questionnaire; CHQ = Child Health Questionnaire Parent Form 28; PHS = Physical Summary score; PSS = Psychosocial Summary score

Notes: Children’ age, gender, IQ level, learning disability, body weight status, chronic disease history, scores on baseline and parents’ marital status, family monthly income, government CSSA status, and mothers’ age, body weight status, employment status, education level, chronic disease status were adjusted for in the multiple linear regressions. **p* value <0.05 is statistically significant

Supplementary Table 11. Sensitivity analysis of the association between Health Empowerment Program and change in outcomes after 5-year follow-up using generalized estimating equations

|  | B (95% CI) | *p*-value for *β* | *Wald chi^2^* | *p-value for Wald test* |  |
| --- | --- | --- | --- | --- | --- |
| **SDQ** |  |  |  |  |  |
| Emotional symptoms | -0.11( -0.53, 0.30) | 0.591 | 232.59 | <.001* |  |
| Conduct problems | -0.66( -1.00, -0.32) | <.001* | 274.62 | <.001* |  |
| Hyperactivity inattention | -0.68( -1.15, -0.21) | 0.005* | 129.21 | <.001* |  |
| Peer problem | -0.33( -0.76, 0.09) | 0.127 | 221.87 | <.001* |  |
| Prosocial behavior | 0.58(0.07, 1.08) | 0.026* | 144.57 | <.001* |  |
| Total difficulties | -1.90( -3.08, -0.71) | 0.002* | 151.85 | <.001* |  |
| **CHQ** |  |  |  |  |  |
| PHS | 0.60( -1.54, 2.73) | 0.582 | 133.97 | <.001* |  |
| PSS | 2.86(0.64, 5.07) | 0.012* | 178.63 | <.001* |  |

SDQ= Strengths and Difficulties Questionnaire; CHQ = Child Health Questionnaire Parent Form 28; PHS = Physical Summary score; PSS = Psychosocial Summary score

Notes: Children’ age, gender, IQ level, learning disability, body weight status, chronic disease history, scores on baseline and parents’ marital status, family monthly income, government CSSA status were adjusted for in the analysis. **p* value <0.05 is statistically significant.
